# Supplementary material for: Rotational orientation control of a ground state ortho-H2 dissociation on a metal surface
Source: Nat Commun. 2025 May 19;16:4625. doi: 10.1038/s41467-025-59928-3 (PMC12089284; doi:10.1038/s41467-025-59928-3)
Supplement: Supplementary file 1 — Supplementary Information [file 41467_2025_59928_MOESM1_ESM.pdf]

Supplementary Information for:

Rotational orientation control of ground-state ortho-H_2 dissociation on a metal surface.

Authors: H. Chadwick[1], G. Zhang[1], C. J. Baker[1], P. L. Smith[1] and G. Alexandrowicz[1*]

Affiliations:

[1]Department of Chemistry, Faculty of Science and Engineering, Swansea University, Swansea SA2 8PP, UK.

*Corresponding author: g.n.alexandrowicz@swansea.ac.uk

The PDF file includes:

Suppl. Note 1. Determination of the molecular beam velocities and control currents.

Suppl. Note 2. Estimation of the coverage during the sticking measurements

Suppl. Note 3. Estimation of the ratio of the 'helicopter' and 'cartwheel' sticking coefficients

Suppl. Figs. 1 to 6

Suppl. Note 1. Determination of the molecular beam velocities and control currents

For the data measured at a nozzle temperature (T_N) of 84K, 106K and 149K data measured with a pure H_2 beam (figures 2 and 3 in the main manuscript), the velocities were obtained from full interferometer measurements where a second hexapole positioned before the detector is used to perform state-to-state scattering measurements[1]. The value of the current in the solenoid of the second arm of the apparatus was set to I_2 = 0A. The measured oscillation curves, presented in the top panel of supplementary figure 2, were fit using a procedure described previously[2] where parameters including the central velocity and full width at half maximum (FWHM) of the velocity distribution (which is modelled as a gaussian) are allowed to vary. A comparison of the velocity distributions that were obtained are presented in the bottom panel of supplementary figure 2. For the T_N = 84K measurement, the central velocity is 1344ms^{-1} with a FWHM of

6.9%, the T_N = 106K velocity is 1513ms^{-1} with a FWHM of 7%, and at T_N = 149K the velocity is 1786ms^{-1} with a FWHM of 7.8%.

A slightly different procedure was used for the 10% He in H_2 reactivity measurement, where seeding the beam with helium leads to a small reduction of the mean H_2 velocity. In this case, the oscillations in the short flux detection measurement that was made to determine the optimal control currents to use in the reactivity measurement were scaled to find the velocity that best matched the oscillation frequency of the signal. The top panel of supplementary figure 3 presents the flux detection measurement measured for a pure H_2 beam (black circles) and the 10% He in H_2 mix (red crosses), and the bottom panel the resulting velocity distributions. This gave a central velocity of 1410ms^{-1} and FWHM of 8% for the 10% He seeded H_2 beam.

The slight reduction in the H_2 velocity when the beam is seeded with He meant that the values of the magnetic field which maximise the 'helicopter' and 'cartwheel' populations in the two beams need to be shifted by 0.0013A with respect to the optimal values for the pure beam. Calculations were performed analogously to those shown in the bottom panel of figure 2 of the main manuscript for the velocity distribution obtained for the seeded beam, to determine how the m_J state populations changed as a function of magnetic field for this different velocity distribution. The results of these calculations are presented in supplementary figure 4.

<u>Suppl. Note 2. Estimation of the coverage during the sticking measurements</u>

To determine the equilibrium coverage during the reactivity measurements presented in figure 4 of the main manuscript, we monitored the transient change in the specularly scattered helium intensity when a clean surface is exposed to the beam. We used the same conditions for the molecular beam which were used in the rotationally controlled sticking measurements (T_N = 106K, 10% He in H_2 mixture and a surface temperature of 375K). A separation valve along the beam line was opened abruptly to allow the molecular beam to hit the surface and the drop in helium signal due to the adsorption of H atoms was recorded, until it had plateaued, after which the separation valve was then closed. The results of this measurement are shown in supplementary figure 5.

Exposing the surface to the mixed beam leads to a decay of the helium signal to 62.5±1.5% of its value before H atoms were adsorbed on the surface. To relate this decay to the H atom coverage, we performed a second set of experiments at a low enough temperature where desorption is negligible (220K) and we can follow the growth of the surface layer up to the formation of an ordered structure at a coverage of 0.5ML[3]. The layer was grown by back-filling the UHV chamber with a H_2 pressure of 5×10^{-7}mbar and monitoring how the scattering intensity of a molecular beam of He changes as a function of hydrogen dose. This is shown in the top panel of supplementary figure 6 for two repeat measurements where scattering into the specular channel was monitored (black solid and red dotted lines), and one where the scattering into the ½ order diffraction channel was measured (blue dashed line). To convert from hydrogen dose (ε) to hydrogen coverage (θ), it was assumed that the sticking coefficient, S(θ), decreased linearly from 0.04 to 0 for coverages between 0 and 0.5 monolayer. i.e., $S(\theta) = \frac{d\theta}{d\varepsilon} = 0.08(0.5 - \theta)$. Previous studies have shown that the initial sticking coefficient is on this order[4–7] for the beam energies we used in this study. The differential equation for $\frac{d\theta}{d\varepsilon}$ gives us the coverage as a function of dose ($\theta = 0.5(1 - \exp(-0.08\varepsilon))$), which can then be used to convert the H_2 dose in the back-filling

measurement to coverage, from which the dependence of the helium intensity on hydrogen coverage immediately follows. The result of this is presented in the bottom panel of supplementary figure 6 for each of the intensity measurements presented in the top panel, where the intensity of the measured signals, $\boldsymbol{\Lambda}$, have been normalised to the maximum intensity, $\boldsymbol{\Lambda_0}$. This conversion produces a maximum in the ½ order diffraction peak scattered intensity at a coverage of approximately 0.5 monolayers, in agreement with that found in previous work[3].

Assuming that the drop in the relative signal intensity due to adding H atoms on the surface is independent of the surface temperature, we can use the 37.5% intensity drop from the first measurement to estimate the hydrogen coverage on the surface as 12±0.5% of a monolayer, as shown by the grey line in the bottom panel of supplementary figure 6. Whilst this is a relatively crude estimation, it is in reasonable agreement with previously obtained values[5] at similar surface temperatures and H_2 pressures.

<u>Suppl. Note 3. Estimation of the ratio of the 'helicopter' and 'cartwheel' sticking coefficent</u>

The relatively low equilibrium coverage the sticking measurement was performed at allows us to use a simple linear model to relate the attenuation of the helium signal to the adsorbate coverage[8], $\frac{\boldsymbol{\Lambda}}{\boldsymbol{\Lambda_0}} = \mathbf{1} - \boldsymbol{\beta}\theta$, where $\boldsymbol{\beta}$ is a constant (see bottom panel of supplementary figure 6). The coverages from the reactivity measurement can then be calculated from the values of $\frac{\boldsymbol{\Lambda}}{\boldsymbol{\Lambda_0}}$ that were obtained as $\theta = (\mathbf{1} - \frac{\boldsymbol{\Lambda}}{\boldsymbol{\Lambda_0}})/\boldsymbol{\beta}$. Taking the ratio of the two coverages at the two solenoid current values which we will denote I_{1a} and I_{1b}, and normalising the signal such that $\boldsymbol{\Lambda_0} = \mathbf{1}$ gives $\frac{\theta(I_{1a})}{\theta(I_{1b})} = \frac{\mathbf{1}-\boldsymbol{\Lambda}(I_{1a})}{\mathbf{1}-\boldsymbol{\Lambda}(I_{1b})}$. The values of $\boldsymbol{\Lambda}(I_{1a})$ and $\boldsymbol{\Lambda}(I_{1b})$ can be found from applying the ±0.5% modulation seen in figure 4c to the attenuation of the helium signal when we do not modulate the populations ($\frac{\boldsymbol{\Lambda}}{\boldsymbol{\Lambda_0}} = \mathbf{0.625}$, shown in supplementary figure 5) resulting in $\frac{\boldsymbol{\theta}(\boldsymbol{I_{1a}})}{\boldsymbol{\theta}(\boldsymbol{I_{1b}})} = \mathbf{0.98}$.

On the other hand the coverage at the two control currents can also be calculated as the product of the flux of the beam and the sticking coefficients of the different states, i.e., $\theta(\boldsymbol{I_1}) = \mathrm{F}[\boldsymbol{S_H}\boldsymbol{N_{-1}}(I_1) + \boldsymbol{S_H}\boldsymbol{N_1}(I_1) + \boldsymbol{S_c}\boldsymbol{N_0}(I_1)]$ where $\boldsymbol{N}_{m_J}(I_1)$ is the proportion of the beam in a given m_J state and at a given solenoid current (I_1), $\boldsymbol{S_H}$ is the sticking probability for helicopter (m_J= ±1) molecules, $\boldsymbol{S_c}$ is the sticking probability for cartwheel (m_J= 0) molecules and F is the flux of the molecular beam. Defining a ratio for the sticking coefficients of helicopter and cartwheel molecules, $\boldsymbol{\alpha}' = \frac{\boldsymbol{S_H}}{\boldsymbol{S_C}}$, we can equate the two expressions for the coverage ratio $\frac{\boldsymbol{\alpha}'[\boldsymbol{N_{-1}}(I_{1a})+\boldsymbol{N_1}(I_{1a})]+\boldsymbol{N_0}(I_{1a})}{\boldsymbol{\alpha}'[\boldsymbol{N_{-1}}(I_{1b})+\boldsymbol{N_1}(I_{1b})]+\boldsymbol{N_0}(I_{1b})} = \frac{\boldsymbol{\theta}(I_{1a})}{\boldsymbol{\theta}(I_{1b})} = \mathbf{0.98}$. Using the calculated populations at the two currents (supplementary figure 4) we obtain that the ratio for the sticking coefficients of helicopter to cartwheel molecules is $\boldsymbol{\alpha}' = \mathbf{1.2}$.

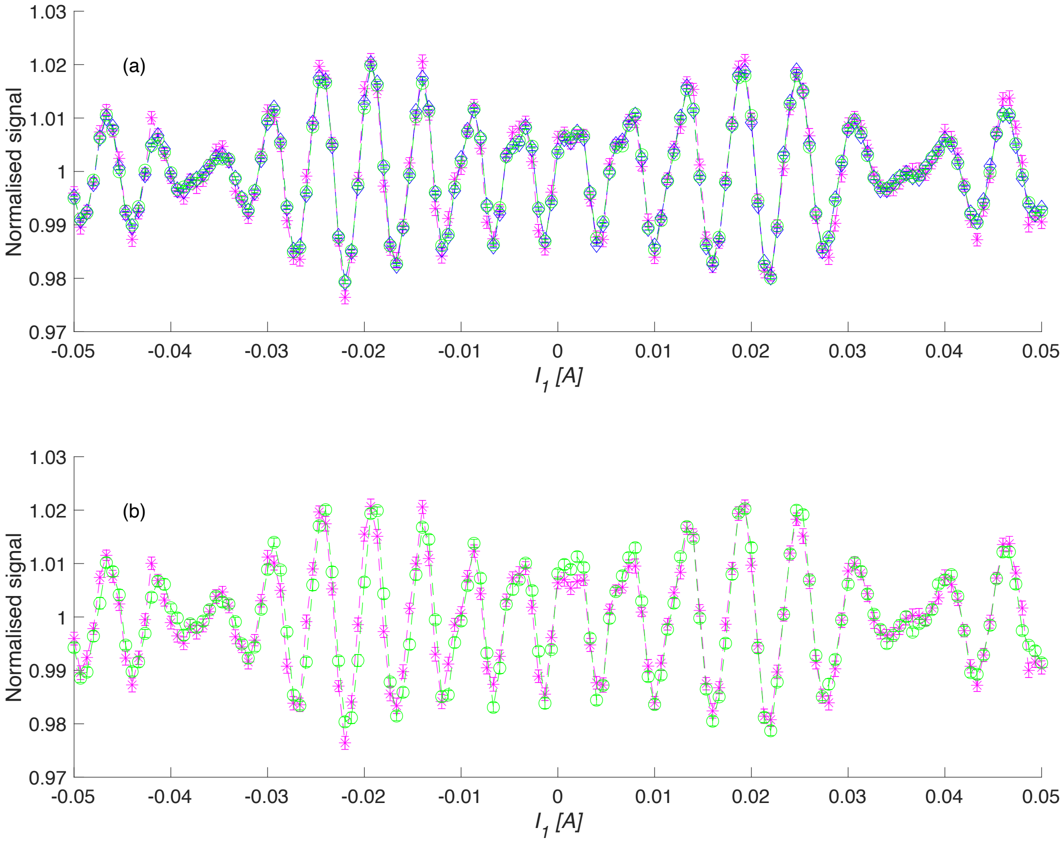

Suppl. Fig 1. **(a)** Comparison of flux detection measurements performed at different crystal azimuths for a 500K surface temperature and a 106K nozzle temperature. The magenta asterisk and green circle markers show the results measured along the $[\mathbf{11\bar{2}}]$ and $[\mathbf{10\bar{1}}]$ directions respectively, whereas the results when measuring in between these two azimuths is plotted using the blue diamond markers. All of the results are identical within the experimental uncertainties. The error bars correspond to the standard error from repeated I_1 scans. **(b)** Comparison of flux detection measurements performed at two different temperatures where the surface is still reactive. The magenta asterisk and green circle markers show the results for surface temperatures of 500K and 375K respectively, using the same nozzle temperature (106K) and measuring along the same crystal azimuth ($[\mathbf{11\bar{2}}]$). The results are identical within the experimental uncertainties. The error bars correspond to the standard error from repeated I_1 scans. Source data for both panels are provided as a Source Data file.

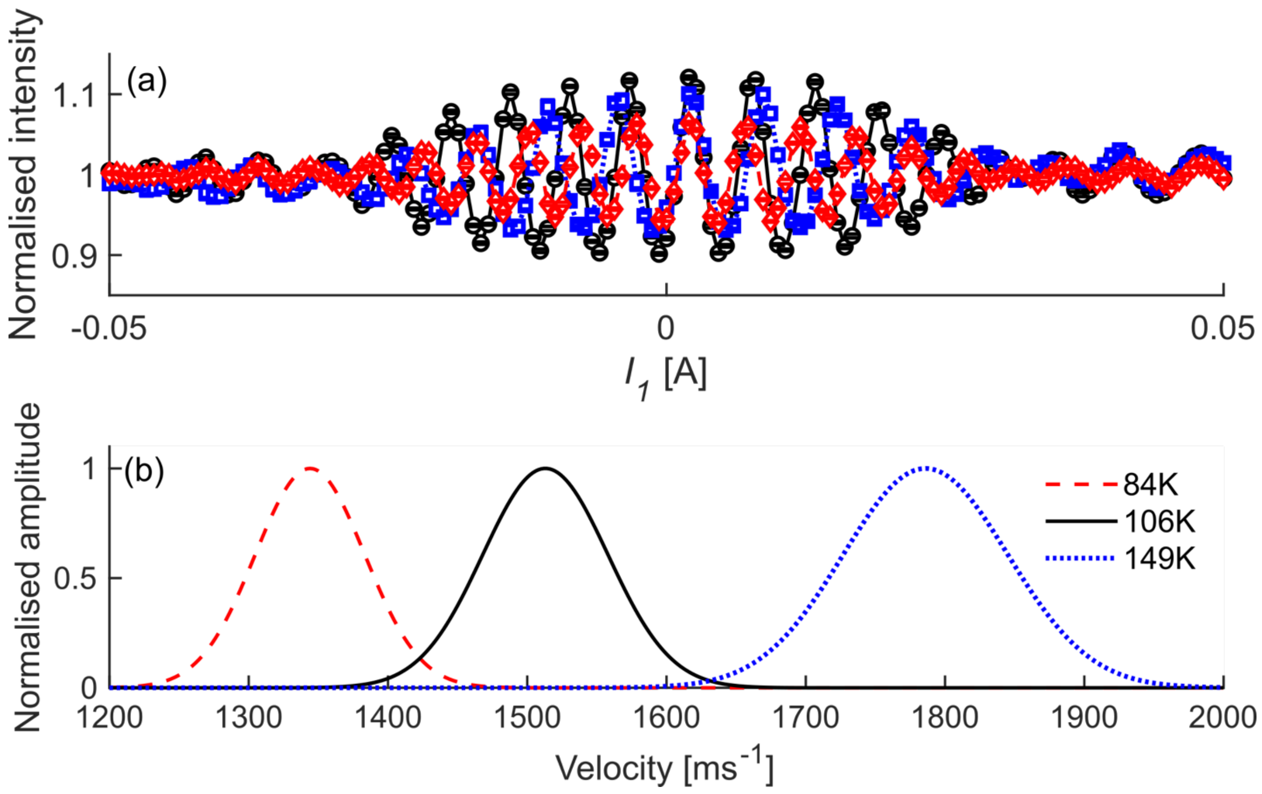

Suppl. Fig 2. (a) Comparison of full-interferometer oscillation curve measurements performed at different nozzle temperatures. The red diamond markers correspond to a nozzle temperature of 84K, the black circles to 106K and the blue squares to 149K. The surface temperature was 500K, and the second solenoid current 0A. The error bars correspond to the standard error from repeated I_1 scans. **(b)** Comparison of the velocity distributions for the different nozzle temperatures. The red dashed line corresponds to the velocity distribution obtained by fitting the red oscillation curve in panel (a) measured at a nozzle temperature of 84K, the black solid line to 106K and the blue dotted line to 149K. Source data for both panels are provided as a Source Data file.

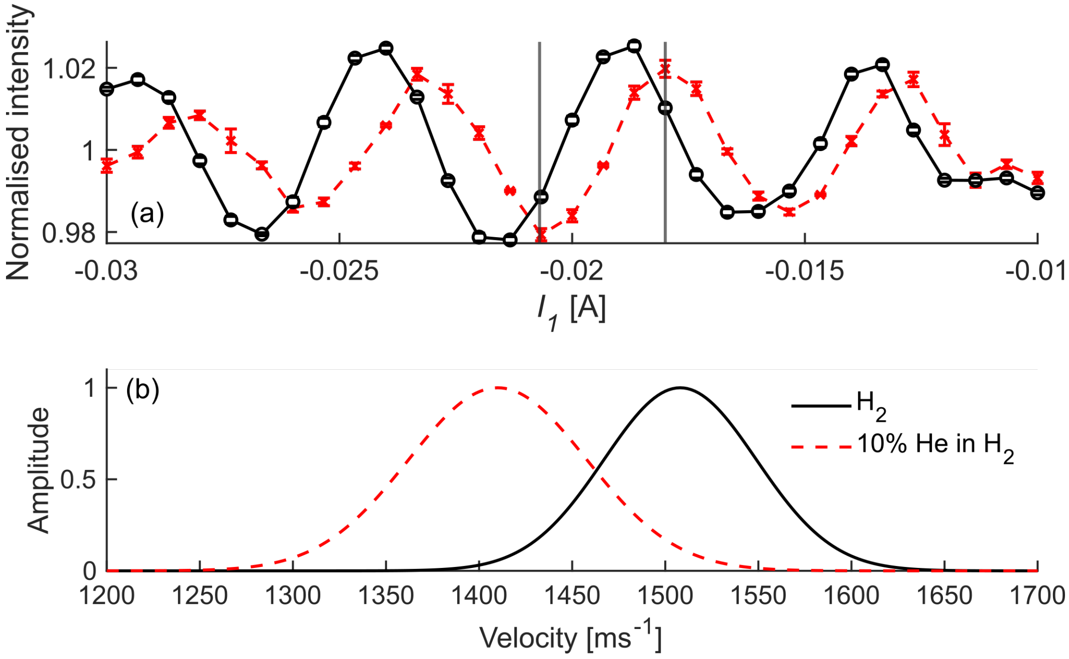

Suppl. Fig. 3. **(a)** Comparison of flux-detection oscillation curves measured for two different incident molecular beam conditions. The black circle markers correspond to a pure H_2 molecular beam, and the red crosses to the 10% He in H_2 molecular beam, and lines have been added to guide the eye. The error bars correspond to the standard error from repeated I_1 scans. The vertical grey lines show the currents used for the measurement presented in figure 4 of the main manuscript. **(b)** Comparison of the molecular beam velocity distributions obtained for the pure H_2 molecular beam (black solid line) and the 10% He in H_2 molecular beam (red dashed line). Source data for both panels are provided as a Source Data file.

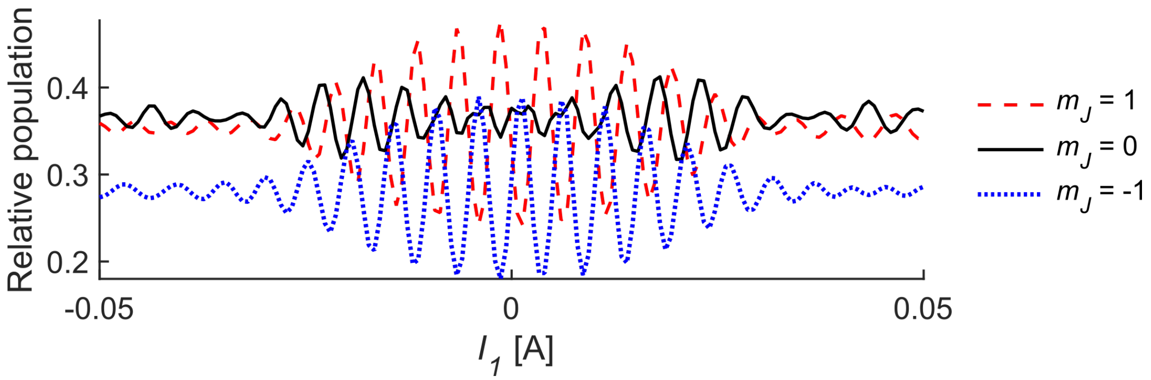

Suppl. Fig. 4. Calculated $m_J = 1$ (red dashed line), $m_J = 0$ (black solid line) and $m_J = -1$ (blue dotted line) state populations at the surface as a function of solenoid current, using the reduced velocity for H_2 when 10% He is added to the beam. The surface normal was used as the quantisation axis. Source data are provided as a Source Data file.

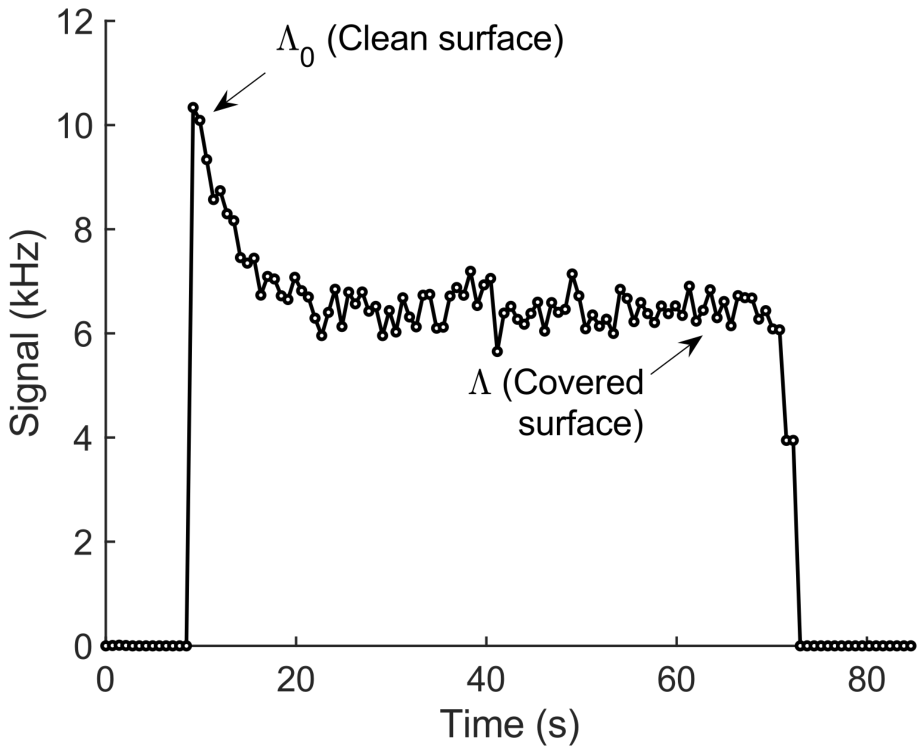

Suppl. Fig. 5. Specularly scattered ^{4}He signal obtained when opening and closing a separation valve for a 10% He in H_2 mix colliding with a Ni(111) surface held at a surface temperature of 375K. Source data are provided as a Source Data file.

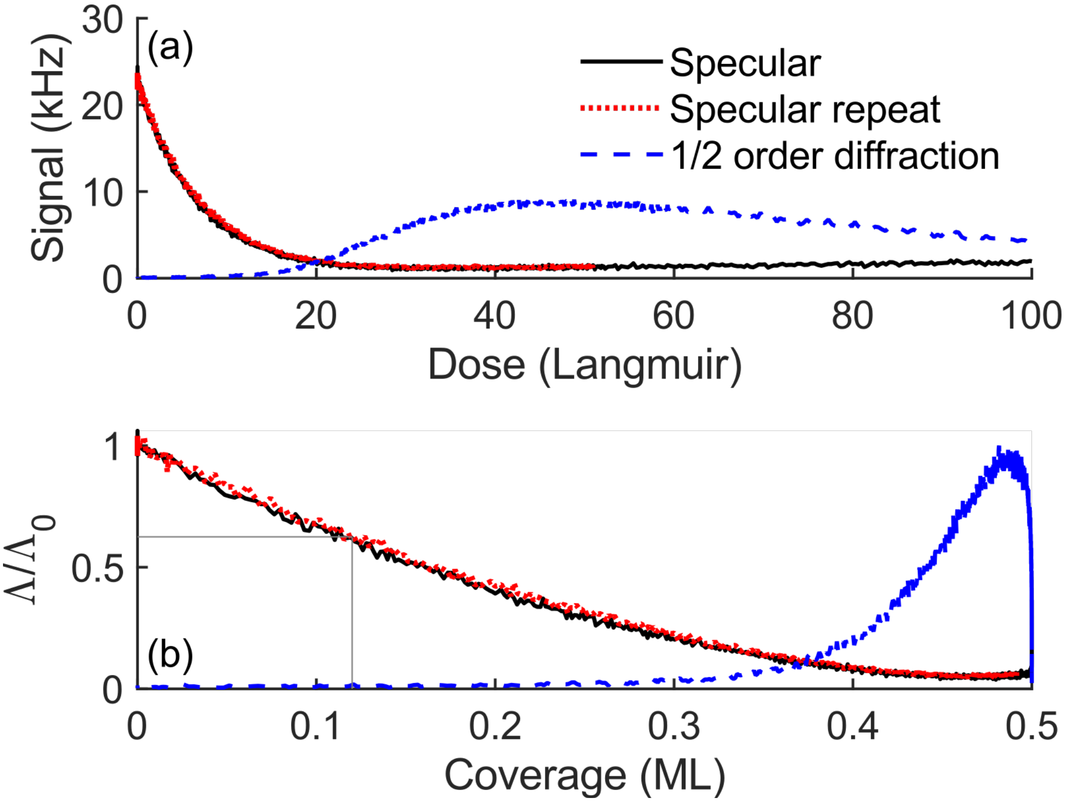

Suppl. Fig 6. (a) Comparison of scattered helium intensity as a function of hydrogen dose during various uptake measurements performed by backfilling the UHV chamber with H_2 at a pressure of 5x10^{-7}mbar at a surface temperature of 220K. The black solid and red dotted lines both show a measurement monitoring the ^{4}He scattering intensity on specular, and the blue dashed line the ^{4}He intensity scattered into a ½ order diffraction peak. **(b)** Normalised ($\boldsymbol{\Lambda/\Lambda_0}$) ^{4}He scattered signal as a function of H coverage (θ) on the surface obtained from the measurements shown in panel (a). The two specular measurements are again shown as a black solid and red dotted line, and the diffraction peak a blue dashed line. See text for details. Source data for both panels are provided as a Source Data file.

Supplementary references

1. Alkoby, Y. *et al.* Setting benchmarks for modelling gas–surface interactions using coherent control of rotational orientation states. *Nat. comm.* **11**, 3110 (2020).
2. Chadwick, H. & Alexandrowicz, G. Temperature dependent stereodynamics in surface scattering measured through subtle changes in the molecular wave function. *Faraday Discussions* (2024).
3. Christmann, K., Behm, R., Ertl, G., Van Hove, M. & Weinberg, W. Chemisorption geometry of hydrogen on Ni (111): Order and disorder. *The Journal of Chemical Physics* **70**, 4168–4184 (1979).
4. Resch, C. *et al.* Dynamics of hydrogen adsorption on promoter-and inhibitor-modified nickel surfaces. *Chemical physics* **177**, 421–431 (1993).
5. Rendulic, K. D. & Winkler, A. Adsorption isotherms obtained from thermal desorption data for the system H_2/Ni(111). *The Journal of Chemical Physics* **79**, 5151–5155 (1983).
6. Steinrück, H. P., Rendulic, K. D. & Winkler, A. The sticking coefficient of H2 on Ni(111) as a function of particle energy and angle of incidence: A test of detailed balancing. *Surface Science* **154**, 99–108 (1985).
7. Winkler, A. & Rendulic, K. D. Adsorption kinetics for hydrogen adsorption on nickel and coadsorption of hydrogen and oxygen. *Surface Science* **118**, 19–31 (1982).
8. Farias, D. & Rieder, K.-H. Atomic beam diffraction from solid surfaces. *Reports on Progress in Physics* **61**, 1575–1664 (1998).
